# The Caribbean and Mesoamerica Biogeochemical Isotope Overview (CAMBIO)

**DOI:** 10.1038/s41597-024-03167-6

**Published:** 2024-04-08

**Authors:** Claire E. Ebert, Sean W. Hixon, Gina M. Buckley, Richard J. George, Sofía I. Pacheco-Fores, Juan Manuel Palomo, Ashley E. Sharpe, Óscar R. Solís-Torres, J. Britt Davis, Ricardo Fernandes, Douglas J. Kennett

**Affiliations:** 1 Department of Anthropology, University of Pittsburgh, 3302 WWPH, Pittsburgh, PA 15260 USA; 2 Department of Integrative Biology, Oregon State University, 4575 SW Research Way, Corvallis, OR 97331 USA; 3 Max Planck Institute of Geoanthropology, Kahlaische Strasse 10, D-07745 Jena, Germany; 4 Interdisciplinary Center for Archaeology and the Evolution of Human Behaviour (ICArEHB), Faculdade das Ciências Humanas e Sociais, Universidade do Algarve, Campus de Gambelas, 8005-139, Faro, Portugal; 5 Department of Anthropology, University of California, Santa Barbara, Santa Barbara, CA 93106 USA; 6 Anthropology Department, Hamline University, 1536 Hewitt Avenue, Saint Paul, MN 55104 USA; 7 Department of Anthropology, University of Arizona, 1009 E South Campus Dr, Tucson, AZ 85721 USA; 8 Center for Tropical Paleoecology and Archaeology, Smithsonian Tropical Research Institute, Luis Clement Avenue, Bldg. 401 Tupper, Ancon, Panama Republic of Panama; 9 Instituto Nacional de Antropología e Historia (INAH), Moneda 16, Col. Centro, Alcaldía Cuauhtémoc, 06060 Ciudad de México, México; 10 School of Human Evolution and Social Change, Arizona State University, 900 S. Cady Mall, Tempe, AZ 85281 USA; 11 Department of Bioarchaeology, Faculty of Archaeology, University of Warsaw, Krakowskie Przedmieście 26/28, 00-927 Warsaw, Poland; 12 Climate Change and History Research Initiative, Princeton University, 129 Dickinson Hall, Princeton, NJ 08544-1017 USA; 13 Arne Faculty of Arts, Masaryk University, Nováka 1, 602 00 Brno, Czech Republic

**Keywords:** Archaeology, Biological anthropology

## Abstract

The Caribbean & Mesoamerica Biogeochemical Isotope Overview (CAMBIO) is an archaeological data community designed to integrate published biogeochemical data from the Caribbean, Mesoamerica, and southern Central America to address questions about dynamic interactions among humans, animals, and the environment in the region over the past 10,000 years. Here we present the CAMBIO human dataset, which consists of more than 16,000 isotopic measurements from human skeletal tissue samples (δ^13^C, δ^15^N, δ^34^S, δ^18^O, ^87^Sr/^86^Sr, ^206/204^Pb, ^207/204^Pb, ^208/204^Pb, ^207/206^Pb) from 290 archaeological sites dating between 7000 BC to modern times. The open-access dataset also includes detailed chronological, contextual, and laboratory/sample preparation information for each measurement. The collated data are deposited on the open-access CAMBIO data community via the Pandora Initiative data platform (https://pandoradata.earth/organization/cambio).

## Background & Summary

The Caribbean, Mesoamerica, and southern Central America are extremely diverse in their geology, ecology, and climate. Archaeological research has nevertheless demonstrated millennia of inter-regional interaction resulting in shared cultural trajectories and the development of common economic, social, and political practices^1–11^. Over the past five decades, isotopic reconstructions of past human diets and mobility in these regions have increased dramatically, providing key insights into the origins of maize agriculture and its intensification, the rise of social complexity and urbanism, and the impacts of European colonization^8,12–20^.

**Fig. 1:**
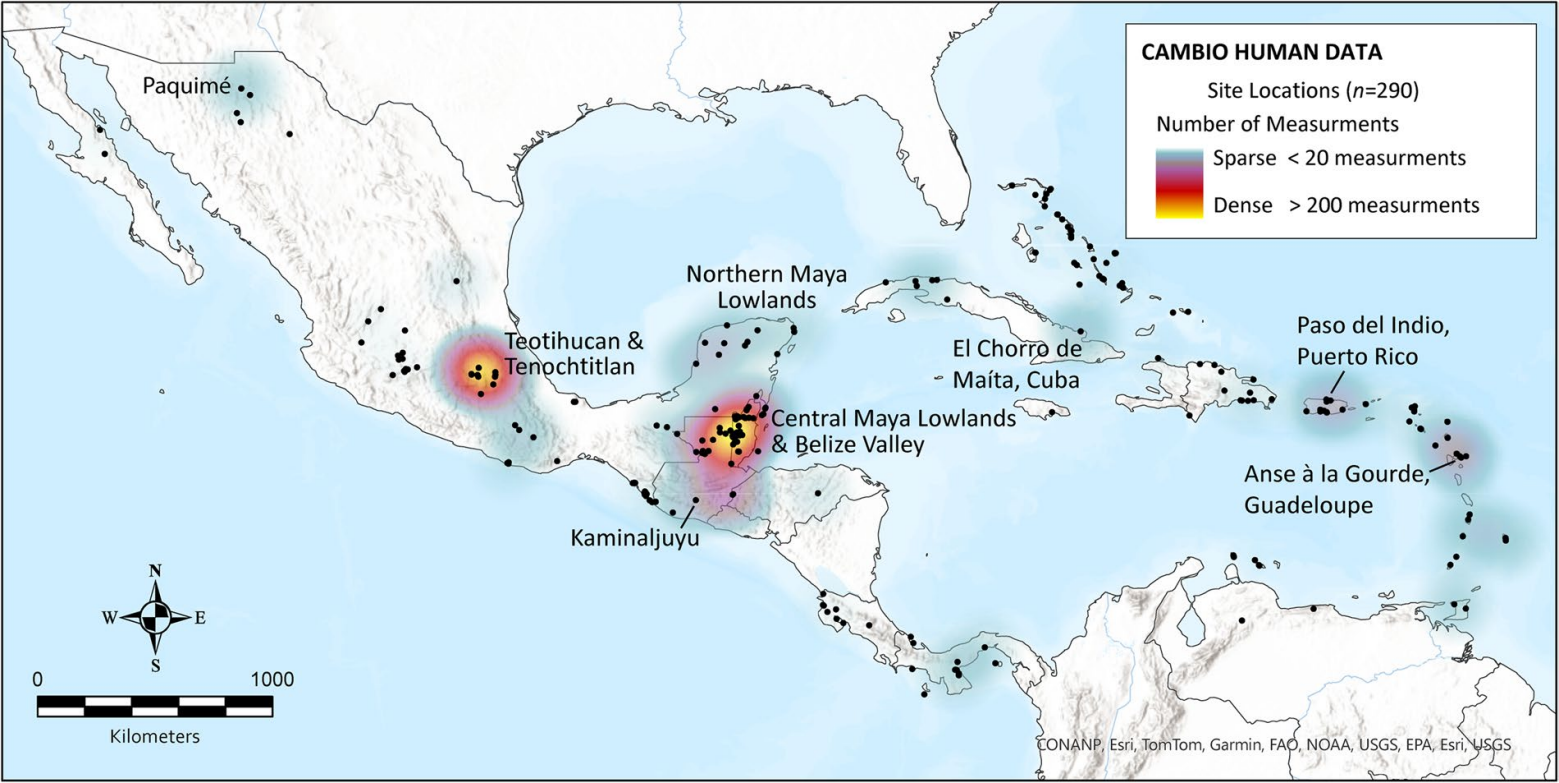
Spatial distributions of human site locations compiled in CAMBIO. Heatmap visualization shows sample size densities of isotopic measurements (>20 measurements). Sites and geographic regions with >200 measurements are labelled. Base map images are the intellectual property of Esri and are used herein under license. Copyright 2023 Esri and its licensors. All rights reserved.

Despite decades of extensive biogeochemical research in Caribbean, Mesoamerican, and Central American archaeology^13,15,17,20^, comparative regional and diachronic syntheses of human diet and mobility have been limited by several factors. First, preservation of skeletal remains is variable in the humid, tropical environments of the region. Laboratory observations estimate that between 30–50% of human skeletal samples are too poorly preserved for many types of biogeochemical analysis (e.g., collagen extraction)^21–23^. Second, sampling bias has resulted in uneven geographic representation throughout the study area (Fig. 1). The highest coverage of isotopic research is in the southern Maya lowlands of Mexico, Guatemala, Belize, and western Honduras^14,18,24^. Substantial research has also focused on several large (100+ individuals), well-studied skeletal assemblages from the Caribbean islands of Cuba, Puerto Rico, and Guadeloupe^13,23,25–27^, and in central Mexico at the city of Teotihuacan^28–33^. Third, a lack of standardization has created inconsistencies in reporting and publication of isotopic datasets. Early studies frequently presented only average isotope values for multiple individuals and infrequently reported sample quality control parameters (e.g., collagen yields and/or atomic C:N ratios^34,35^). Other inconsistencies are related to reporting (or lack thereof) of chronological information and age/sex estimates. For example, scholars have applied different methods for reporting age estimates that are not easily comparable^15^. In other instances, some studies include previously published data without citing the original publication or reporting which variables come from these sources. Finally, and perhaps most importantly, large-scale synthetic isotopic studies from the Caribbean, Mesoamerica, and southern Central America have been prohibited by a lack of open access to published datasets. Most archaeological biogeochemical studies are published in English-language journals and are often inaccessible to our colleagues and communities based in Latin America and the Caribbean due to pay-wall restrictions. Limited data access highlights the academic exclusivity of researchers at universities in the United States, Canada, and Europe^36,37^. The recent availability of multiple large biogeochemical datasets from around the globe demonstrates that isotopic analysis is a powerful tool for understanding aspects of past human lifeways including diet, nutrition, and population movement^38–41^.

The Caribbean & Mesoamerica Biogeochemical Isotope Overview (CAMBIO) is a collaborative effort led by early career researchers in archaeology based in the US, Latin America, and Europe to systematically compile published biogeochemical data from the Caribbean, Mesoamerica, and southern Central America in an open-access and multilingual format (Spanish, French, and English). Importantly, our efforts focus not only on collecting isotopic datasets, but also the inclusion of provenance and chronological information to accompany datasets, facilitating multiple archaeological, bioarchaeological, and paleoenvironmental applications. The total number of *δ*^13^C, *δ*^15^N, *δ*^34^S, *δ*^18^O, ^87^Sr/^86^Sr, and Pb measurements included is currently 16,512 from at least 5,353 individuals recovered from 290 archaeological sites. The temporal coverage of the dataset represents a broad time span, from the Archaic (~7000 BC) to the Colonial/Historic period (~AD 1500–1800).

Most recorded data (~81%) are stable isotope measurements (*δ*^13^C, *δ*^15^N, *δ*^34^S, *δ*^18^O) from bone and dentine collagen (~41.6%), bone bioapatite (~13.1%), enamel bioapatite (22.4%), and bone and enamel phosphate (~3.7%) (Table 1). The remainder of the data consists of radiogenic isotopic measurements, mostly ^87^Sr/^86^Sr measurements from enamel and bone bioapatite (~16.6%). Approximately 1.5% of the dataset includes Pb measurements, reflecting the exploratory nature of Pb analysis to track the origin and movement of human populations in the CAMBIO region^42^. For both ^87^Sr/^86^Sr and Pb, a local or non-local origin was designated based on reporting by the original authors.

**Table 1:** Number of measurements in CAMBIO human database listed by skeletal tissue (“Analyzed Component”) and isotopic measurement type.

Analyzed Component	Stable Isotopes				Radiogenic Isotopes					Total Measurements	Total Database %
	*δ*^13^C	*δ*^15^N	*δ*^34^S	*δ*^18^O	^87^Sr/^86^Sr	^206/204^Pb	^207/204^Pb	^208/204^Pb	^207/206^Pb		
Bone collagen	3165	3067	138	0	0	0	0	0	0	6370	**38.6%**
Dentine collagen	260	228	0	0	0	0	0	0	0	488	**3.0%**
Dentine carbonate	0	0	0	0	5	0	0	0	0	5	**0.0%**
Bone bioapatite	1,391	0	0	771	288	0	0	0	0	2450	**14.8%**
Enamel bioapatite	1492	0	0	2208	2450	117	117	117	88	6589	**39.9%**
Bone phosphate	0	0	0	346	0	0	0	0	0	346	**2.1%**
Enamel phosphate	0	0	0	264	0	0	0	0	0	264	**1.6%**
**Total Measurements**	**6308**	**3295**	**138**	**3589**	**2743**	**117**	**117**	**117**	**88**	**16512**	**100.0%**
**Total Database %**	**38.2%**	**20.0%**	**0.8%**	**21.7%**	**16.6%**	**0.7%**	**0.7%**	**0.7%**	**0.5%**	**100.0%**	

## Methods

Data collection for the CAMBIO human dataset began in May 2021, focusing on three major geographic regions: 1) the Caribbean (islands of the Bahamian Archipelago and the Greater and Lesser Antilles), 2) Mesoamerica (modern countries of Mexico, Guatemala, Belize, Honduras, and El Salvador), and 3) southern Central America (modern countries of Nicaragua, Costa Rica, and Panama). A very small number of measurements were also included from northern South America (coastal Venezuela) based on reported cultural affiliation with pre-contact Caribbean groups. The dataset was compiled from published sources including journal articles, books and book chapters, conference proceedings, publicly available academic theses, and archaeological reports in both English and Spanish. Studies were obtained through scientific search engines (e.g., Web of Science, Google Scholar) and online library searches. In cases where datasets could not be obtained online, we contacted the original authors who generously shared their publications. Resources reporting human stable isotope data as of March 2024 are included in the inaugural version of CAMBIO. The dataset in its current form, however, is not completely exhaustive, and will be regularly updated following publication of new studies or the location of previously published studies not yet integrated into the dataset.

**Fig. 2:**
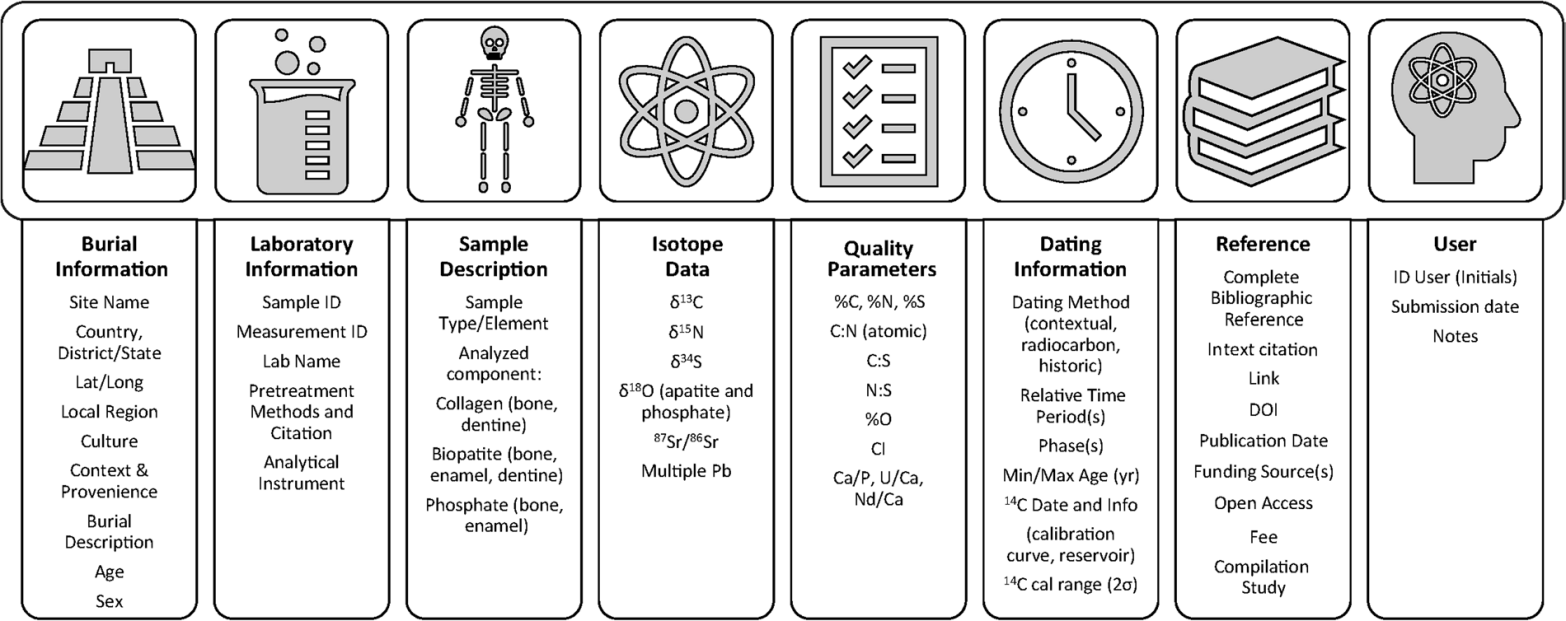
CAMBIO human database structure.

The structure of the CAMBIO human dataset is depicted in Figure 2 and described in detail in the dataset’s accompanying metadata file. The CAMBIO dataset is organized according to a series of nested descriptive fields. Each entry has a unique numerical Entry ID. Entries are listed by skeletal tissue type (“Analyzed component”): bone and dentine collagen; dentine carbonate; bone and enamel bioapatite; and bone and enamel phosphate. Isotopic measurements (*δ*^13^C, *δ*^15^N, *δ*^34^S, *δ*^18^O, ^87^Sr/^86^Sr, ^206/204^Pb, ^207/204^Pb, ^208/204^Pb, ^207/206^Pb) are then reported based on these tissue types. A single individual or burial, therefore, may have multiple entries in CAMBIO if multiple tissues were analyzed (e.g., separate entries from bone collagen and bone bioapatite isotope values). They may also have multiple entries if different elements were sampled (e.g., multiple bones and/or teeth from the same individual), or if samples were incremental (e.g., tooth increments) or duplicated. This data structure was chosen to more easily address research questions that can be evaluated by specific skeletal tissue types or isotopic systems. The approach also facilitates identification of measurements from different elements that might signal changes to diet or movement throughout a single individual’s life. Entries are tied together by burial identification numbers, if assigned by the original investigators, or sample numbers when burial numbers were not reported. Variations of burial/sample numbers were standardized so that entries for the same individual could be correlated, and to eliminate duplicate entries with identical elemental data.

Burial and site information in CAMBIO comprise both spatial and descriptive attributes. Site locations for each entry are reported as decimal degrees (“Latitude”; “Longitude”) relative to the WGS84 spatial reference system. Coordinates are designated as either “reported” or “estimated”. Reported coordinates are those listed in the original publication. When not available, coordinates were estimated by either locating sites or georeferencing maps from the original publications in Google Earth. Spatial coordinates for Maya lowland sites were also derived from the Electronic Atlas of Ancient Maya Sites^43^. Local regions (e.g., Maya Lowlands, Greater Antilles) and cultural affiliations (e.g., Mixtec, Aztec, Lucayan Taino) are also included for each entry when described in the source publication. Other burial information recorded in CAMBIO includes provenience data including a description of the site, structure, excavation unit, level, and/or other information about the location of the burial. A description of pathology was also included if reported in the source publication along with isotopic data. Because researchers use several different methods for age estimation to answer different research questions^15,44^, age estimates are listed as those reported in the source publication, with minimum and maximum age in years, if reported. A second age category (“Age 2”) with adult (18+ yrs.) and sub-adult (0–18 yrs.) designations was also assigned to facilitate data comparisons between broader age cohorts.

The CAMBIO database also includes laboratory and sample preparation information for each measurement. This includes the name of the lab where samples were processed and/or measured, a description of pretreatment protocols, and mass spectrometry instrumentation information. Isotopists are continuously investigating the impacts of different pretreatment protocols on isotope values^45^. For collagen extraction, laboratories use various chemical protocols (e.g., NaOH rinse) and mechanical techniques (e.g., ultrafiltration, or the modified version of the Longin method^46^) to remove exogenous contaminants, with differing impacts on collagen yields and quality^47–51^. More recently, amino acids have been extracted and purified using XAD resin column chromatography^52,53^. This has greatly improved the success rate in obtaining carbon and nitrogen isotopic data from degraded bone samples^21,52,54,55^. Potential complications also exist in the removal of organics from bone and enamel bioapatite before carbonate and phosphate analysis^56–62^. Following best practice recommendations for reporting in archaeological isotopic studies^63^, CAMBIO lists a short description of each sample pretreatment protocol and citation for pretreatment protocols when reported in the original publication.

**Fig. 3:**
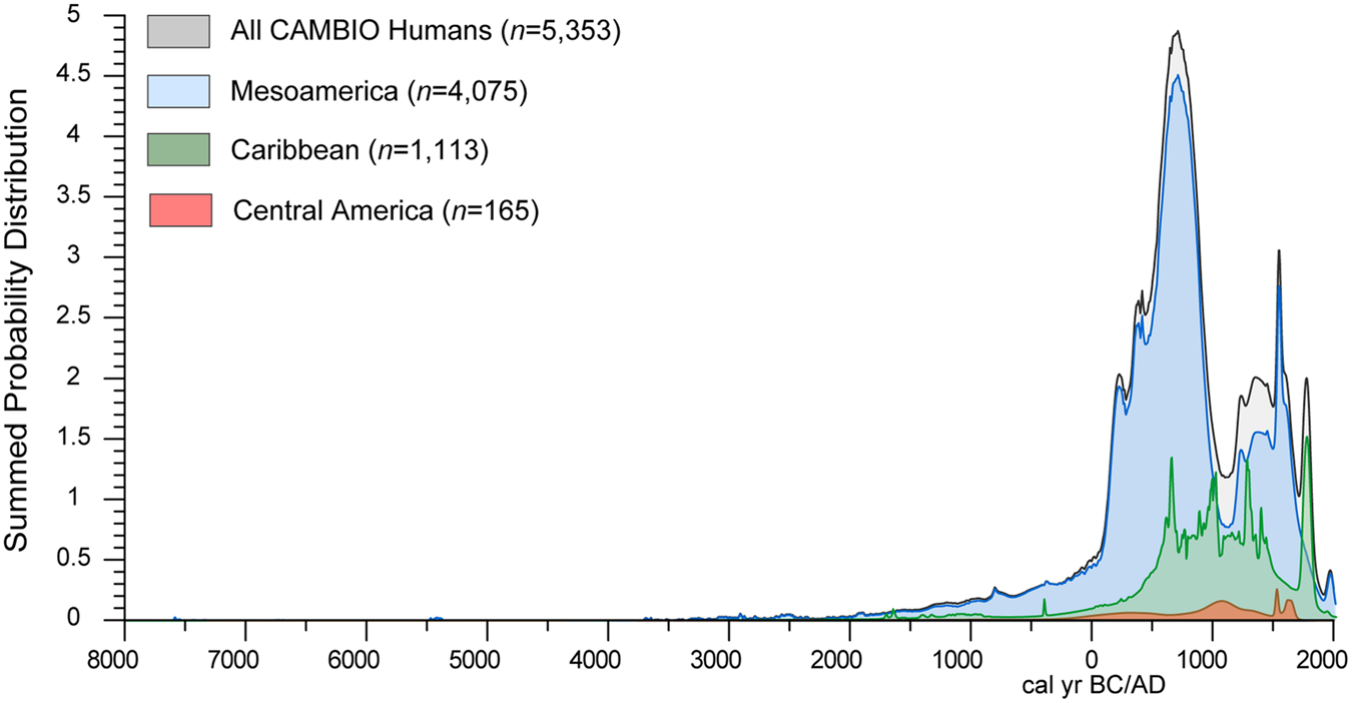
Summed probability distribution of all human individuals (n = 5,353) in the CAMBIO dataset represented by major geographic region. AMS ^14^C dates were calibrated using methods described in text. Contextually dated samples were modelled as a uniform distribution based on minimum and maximum assigned chronological values. Graph was produced using OxCal v. 4.4.3. and edited in Adobe Illustrator v27.9.

Temporal assignments (“Min date”; “Max date” in years BC/AD) for each measurement were based on radiocarbon dates when available, but were primarily assigned using contextual, epigraphic, and historic documents as listed in the original publications. Relative time periods and site-specific archaeological phase designations are also listed when available. All reported radiocarbon measurements in the database are listed by “^14^C date lab number” (if reported) along with the conventional ^14^C date (in cal yr BP) and error. Radiocarbon dates listed in CAMBIO were calibrated in the calibration software OxCal v.4.4^64^ primarily using the IntCal20 calibration curve^65^. Temporal assignments, however, followed calibration procedures reported in the original study. For example, a mixed IntCal20/Marine20^66^ or local freshwater or marine reservoir correction (ΔR) was used if reported, and is listed in the “Curve used for Calibration” and “^14^C reservoir effect” categories in the dataset, respectively. We report all calibrated ^14^C dates at the 2-sigma range. Figure 3 shows an estimate for the chronological distribution of the individuals with isotopic measurements based on both ^14^C and relative temporal assignments (i.e., a summed probability distribution). Samples primarily fall between ~AD 100–1000, reflecting a bias in isotopic data collection, which often focuses on the Classic/Epiclassic urban and elite contexts. The dataset includes a small number of modern measurements from forensic case studies to further contextualize archaeological isotopic values.

We also collected information about the accessibility of each study in the dataset. This information is useful for researchers working outside of the United States, Canada, and Europe or those unaffiliated with an academic institution attempting to locate source publications. Accessibility is listed under the “Open Access” category. We also listed the fees associated with accessing the study if it is not open access. Approximately 59% of studies in the CAMBIO human dataset are behind a paywall with an average of $37.45 USD per publication for unaffiliated researchers. A list of funding sources reported in the original publication is included. Most publications (~82%) in the CAMBIO human database received partial or complete financial support from federal (US), state, and/or other national sources (e.g., National Science Foundation (USA), Consejo Nacional de Ciencia y Tecnología (Mexico), Social Sciences and Humanities Research Council of Canada).

## Data Records

The complete CAMBIO human isotopic dataset is available via a single table in.xlsx format (‘cambio_humans_v.1.xlsx’) through the CAMBIO data community within the Pandora Initiative data platform (10.48493/6c2a-r758)^67^. Metadata descriptions and the current dataset bibliography are provided on separate sheets within the file.

## Technical Validation

CAMBIO includes information about quality control parameters in bioarchaeological isotopic studies when available in the source publication. For bone and dentine, these metrics can be used to assess collagen preservation (“%C”; “%N”; “Atomic C:N ratio”; “Atomic C:S ratio”; “Atomic N:S ratio”). Measured values that fall outside the recommended ranges for collagen^34,68^ are included in the database since they are useful for assessing patterns of sample preservation. Other quality control parameters for bone and enamel apatite can be used to document diagenesis and possibly chemical alteration. For ^87^Sr/^86^Sr, this includes Sr ppm, crystallinity index (“CI”) values, Ca/P ratios, U/Ca ratios, and Nd/Ca ratios^69–72^. Studies that lack quality control criteria, or that employ different reference values, are included in CAMBIO and can be filtered by researchers prior to analyses, if desired.

CAMBIO differentiates between *δ*^18^O measurements from phosphate and carbonates based on the “analyzed component” (bone/enamel phosphate vs bone/enamel bioapatite). Phosphate *δ*^18^O values are expressed relative to the VSMOW standard^73^. Most bioapatite carbonate *δ*^18^O measurements are expressed relative to VPDB in the source publication. Therefore, bioapatite carbonate values originally reported relative to VSMOW were converted to VPDB to standardize the dataset^74–76^. Computational details are listed in the “Notes” section of the database. Some studies also applied conversions of bioapatite carbonate VPDB values to compare with drinking water VSMOW baseline values. In these instances, if VPDB values were not reported, our conversations may introduce some error by passing observations through equations multiple times^77,78^. In cases of conversion, we refer researchers to the original publications before conducting additional analyses.

## Usage Notes

The CAMBIO human isotopic data compilation combines isotopic data informative of diet and place of origin with chronological, bioarchaeological, and archaeological and historical information. This dataset provides facilitated investigations of significant archaeological questions about human behavior and cultural developments in the Caribbean, Mesoamerica, and southern Central America over the past 10,000 years. Potential use includes inter-regional, regional, and site-specific syntheses of paleodietary isotopic data (*δ*^13^C, *δ*^15^N, *δ*^34^S) to assess broad patterns of human resource acquisition and subsistence change. For example, the broad spatial scope of the database facilitates studies about the timing for the spread of domesticates like maize (*Zea mays*) from Mesoamerica to the Caribbean and North and South America. Analyses of other isotopic systems (*δ*^18^C, ^87^Sr/^86^Sr, Pb) can also be used to document large-scale population movement. Potential questions of interests include immigration to Classic period cities, including Teotihuacan and lowland Maya kingdoms, and the forced movement of indigenous populations and enslaved Africans during the colonial era.

## Data Availability

No custom code was used to generate or process the data.
